# Bone-Grafting in Relation to Sepsis

**Published:** 1918-03-30

**Authors:** 


### THE MUSEUM AS A TEACHING CENTRE.

### Bone-Grafting in Relation to Sepsis.

THE museum of the Royal College of Surgeons in Lincoln's Inn Fields, founded by John Hunter, has proved itself a centre to which have been drawn for many years valuable and instructive specimens from all quarters of the globe. One of the main reasons for this success has been that the museum has been, not a mere dead exhibition, but a home of living work. Its curators have infused the collection with organisation and enterprise, and have made it a point from which have spread many important contributions to scientific knowledge. Such an institution ought to have a systematic teaching mission, and, if possible, also a regularised scheme of research. Fortunately the direction of the Hunterian Museum has been guided by these principles, and has appreciated the necessity of securing success by the appointment to the curatorship of men possessed of scientific habit and enthusiasm. Here, indeed, has been the factor which has made the museum a living and effective organisation. The present curator, Professor Arthur Keith, has more than justified his appointment, and has shown himself abundantly worthy of the traditions of his office. The material under his charge is immense, and is rich in lessons relative to many modern surgical problems. What is needed is the eye to read these lessons and the capacity to set them in a convincing position at the service of the modern student. Under Professor Keith's influence the museum is sustaining, or even advancing, its long-established reputation.

In his latest series of lectures on "The Anatomical and Physiological Principles Underlying the Treatment of Injuries of Muscles, Joints, and Bones" we find an excellent example of the functions which the properly directed museum may be made to fulfil. Anatomical specimens and preparations are historical records, and this is more especially true of those which are the outcome of deliberate experiment. Studied in series they display the gradual growth of knowledge, and they throw light on the causes of past failures and help to attain subsequent successes. From them, when placed in proper relationship, emerges the doctrine that the extension of new knowledge is a gradual process rather than a sudden revelation, and the doctrine is as true to-day as yesterday. Hence it follows that the worker set upon advancing truth cannot afford to shut his eyes to the accomplishments, or even to the failures, of those who have preceded him. In both he may find hints at the direction he ought to take, if he would succeed in his ambition and would avoid the waste both of time and effort. His criticism has the further virtue of detachment, since he has no personal surgical ambitions to cultivate. None the less, it is informed criticism, and it is supported by reasons which define themselves in no hesitating fashion. His lectures make most fascinating reading, and they have close relations with practical issues which are in debate at the present hour.

We will take in illustration of these qualities the lecture on "Bone-Grafting" fully reported in the *Lancet* of the 9th February. The subject is one which bears directly upon modern surgical work in more than one direction, and, like other developments, it has a history. John Hunter worked at it, and he almost succeeded. Indeed, in individual instances he did succeed. At least, he successfully re-implanted a tooth which had been knocked out, and recognised the conditions which must exist in order that such a replacement may be successful; and his successful transplantation of cocks' spurs to the legs of hens and *vice versâ* is well known. Inevitably the question arises why did Hunter, in spite of his skill and insight, fail to establish a practice which has now become a regular surgical method? Professor Keith gives us a short and sufficient answer. The failure was due to sepsis. It was this malign agency which ruined the brilliant programme that Hunter had conceived. In a word, the enterprise was born out of due season, for of the nature of sepsis and of the means of avoiding it there was at the date in question no knowledge. As wounds then usually became septic the development was considered to be inevitable and in the natural order of things. Provided only the prevalent discharge was abundant and "creamy" it was regarded with complacency and was dignified as "laudable."

It is a leap from Hunter to 1880 when the modern practice of bone-grafting was invented in the Glasgow Royal Infirmary by Macewen, but in the interval the conditions necessary for success had been established. Pasteur had accomplished his famous work, and under this inspiration Lister had established his life-saving surgical methods. Professor Keith suggests that one influence which favoured Macewen's success was that the work was accomplished in the same institution as that in which Lister had worked out his antiseptic system. By this, we imagine, he implies that Macewen knew the principles which underlie this system and could apply them, as distinct from a certain number of surgeons who professed Lister's practice without any real appreciation of its spirit. Another interesting factor in Macewen's success was that the grafts were placed in a situation which exposed them to muscular stresses and strains. Reasoning in advance, it might have been argued that avoidance of these stresses and strains would promote the survival of the transplanted tissue. But Professor Keith is urgent against this view. "Osteoblasts," he tells us, "are the obedient slaves of muscles," and it is under the stimulating influence of the muscles that the bone-builders take on a really successful career. His remarks on the influence of muscular play on bone growth and bone development are amongst the most stimulating parts of his lectures, and we hope to return to them on another occasion.

